# Otosclerosis and Measles: Do Measles Have a Role in Otosclerosis? A Review Article

**DOI:** 10.7759/cureus.9908

**Published:** 2020-08-21

**Authors:** Prem Raj Sagar, Puja Shah, Vijaya Chaitanya Bollampally, Norah Alhumaidi, Bilal Haider Malik

**Affiliations:** 1 Internal Medicine, California Institute of Behavioral Neurosciences & Psychology, Fairfield, USA; 2 Neurological Surgery, California Institute of Behavioral Neurosciences & Psychology, Fairfield, USA

**Keywords:** conductive hearing loss, measles, measles virus, otosclerosis, stapes, tinnitus

## Abstract

Otosclerosis is a common cause of conductive hearing loss which is an autoimmune inflammatory disorder related to abnormal bone remodeling of the human otic capsule that has complex etiopathogenesis attributed to genetics, autoimmunity, viral infection, inflammation, hormonal factor, environmental factor, and disturbed bone metabolism. It has a prevalence of 0.3%-0.4% in Caucasians, which makes up 5-9% of all hearing loss and 18-22% of all conductive hearing loss. This review article aims to study the postulated role of sustained measles virus infection in the etiopathogenesis of otosclerosis, among others. A PubMed search of the related topic identified 97,990 articles. After we applied the inclusion and exclusion criteria, it was determined that 52 articles were relevant, which included 38 observational studies, 13 review articles, and a systematic review. Among them, 33 observational studies, 13 review articles and a systematic review spotted a positive association between persistent measles virus infection and otosclerosis. On the contrary, five observational studies reported no evidence of the association. The majority of the current literature supported the presence of the measles virus component in the otosclerotic stapes samples and its role in the etiopathogenesis of otosclerosis. Measles virus infection may have the amplitude to initiate a pathological process, which in the presence of other factors like autoimmunity and genes plays a significant role in causing otosclerosis. However, other studies have failed to show the presence of the measles virus component in otosclerotic stapes. So, more studies are needed to probe the role of persistent measles virus infection in the etiopathogenesis of otosclerosis.

## Introduction and background

The prevalence of otosclerosis is 0.3%-0.4% in Caucasians, which makes up 5-9% of all hearing loss and 18-22% of all conductive hearing loss. However, this disease affects only 0.03%-0.1% of Africans and Asians [1-5]. It is two to three times more common in females than males [1, 6]. Histologic otosclerosis is present in 8-11% of the autopsy sample, which is higher than the actual prevalence of the disease [1]. Measles is the cause of 100,000 deaths every year, even after the introduction and global use of the measles vaccine. The casualty attributed to measles before the vaccine use was approximately two million deaths annually [7]. The actual measles incidence and mortality are lacking because most of the cases and death occur in developing countries lacking efficient reporting systems [7].

Otosclerosis is an autoimmune inflammatory disorder related to abnormal bone remodeling of the human otic capsule [1]. It causes progressive conductive hearing loss in the bilateral ear, which is due to stapes footplate fixation [1]. The onset of otosclerosis is usually in the third to fifth decade of life. The disease has complex etiopathogenesis attributed to genetics, autoimmunity, viral infection, inflammation, hormonal factor, environmental factor, and disturbed bone metabolism [1]. Out of these, genetic factor plays a significant role in etiology of otosclerosis, mode of inheritance being autosomal dominant with incomplete penetrance [8]. Measles is an ribonucleic acid (RNA) virus belonging to the paramyxoviridae family. It is a highly contagious viral illness that presents clinically with fever, malaise, rash, cough, coryza, and conjunctivitis [7]. The RNA viruses generally have very high mutation rates compared to DNA viruses, because viral RNA polymerases lack the proofreading ability of DNA polymerases. This is one reason why it is difficult to make effective vaccines to prevent diseases caused by RNA viruses.

The role of sustained measles virus infection is postulated in the etiopathogenesis of otosclerosis [9]. Many studies suggest the association while other studies have failed to establish the role of measles in otosclerosis [2, 10-12]. This questions whether persistent measles virus infection is one of the many causes of otosclerosis or their presence is a mere coincidence. If there is a slightest opportunity to learn the true relationship, it will be of great help to the medical field in simplifying one of the many triggers of otosclerosis, also instigating therapeutic value that can be researched for ameliorating otosclerosis. As a result, there will be a decrease in the public health burden of otosclerosis and significant relief for patients whose life is impacted at the psychosocial and professional level due to hearing loss and tinnitus.

This review article aims to determine the relationship between persistent measles virus infection and otosclerosis by reviewing most of the relevant articles on PubMed. This article also focuses to differentiate whether the relationship exhibited in different articles is actually present or merely coincidental by comparing and contrasting the majority of the available articles on this subject.

## Review

Method

Literature was searched in PubMed using regular keywords and Medical Subject Headings (MeSH) keywords and data collection was done. 

Table 1 shows regular keywords and MeSH keywords for the literature search.

**Table 1:** Regular and MeSH keyword for literature search MeSH: Medical Subject Headings

Regular Keyword	Number of results	MeSH Keyword	Number of results
Measles	28,682	Measles	15,231
Tinnitus	13,075	Measles virus	6,406
Measles virus	11,130	Otosclerosis	5,190
Stapes	6,511	Otosclerosis, Measles Virus	42
Otosclerosis	5,811	Otosclerosis, Measles	34
Conductive hearing loss	5,746		
Otosclerosis, Measles	70		
Otosclerosis, Measles virus	62		

Studies were selected after applying the following inclusion criterion: human studies only. No exclusion criteria were set.

Results

Table 2 shows the total number of articles after applying inclusion criterion.

**Table 2:** Total number of articles after applying inclusion criterion MeSH: Medical Subject Headings

Regular Keyword	Number of results	Number of results after applying inclusion criterion	MeSH Keyword	Number of results	Number of results after applying inclusion criterion
Measles	28,682	24,116	Measles	15,231	14,401
Tinnitus	13,075	10,875	Measles virus	6,406	4,952
Measles virus	11,130	8,537	Otosclerosis	5,190	4,748
Stapes	6,511	5,653	Otosclerosis, Measles Virus	42	41
Otosclerosis	5,811	5,151	Otosclerosis, Measles	34	34
Conductive hearing loss	5,746	5,111			
Otosclerosis, Measles	70	68			
Otosclerosis, Measles virus	62	60			

A total of 62 articles were found with keyword otosclerosis and measles virus by regular search in PubMed. Out of which 60 articles fulfilled the inclusion criteria of human studies. Among the 60 articles that were reviewed, 52 articles were relevant for this research topic.

Finally, these 52 articles were selected for review, out of which 38 articles were observational studies, 13 articles were review articles, and one was a systematic review. The maximum sample size in a study was 1,351 and the aggregate sample size was 5,863.

Discussion

Histopathology of otosclerosis

Otosclerosis is related to abnormal bone remodeling of the human otic capsule. Normal bone remodeling is a naturally occurring process in all bones where there is an equilibrium between bone resorption and bone formation by osteoclasts and osteoblasts, respectively [13]. Abnormal remodeling has four phases: it begins with active, lytic phase of bone resorption with the proliferation of blood vessels, osteoclasts, and mononuclear cells; in the second phase, there is a new bone formation; and the third phase is the inactive phase. The final phase is characterized by dysplastic, compact bone with a woven pattern [14]. Otosclerosis mainly affects stapes footplates, it also affects the adjacent structures like pericochlear, perilabyrinthine, oval, and round window [15]. Its histopathological features include focal, osteolytic bone lesions with high cellularity and vascularization [15]. The abnormal bone remodeling leads to ankylosis of stapes footplates causing conductive hearing loss among other clinical manifestations.

Genetics in otosclerosis

There is a greater incidence of otosclerosis in certain families than in the general population which suggests the role of genes and heredity in its etiopathogenesis [16]. Autosomal dominance with incomplete penetrance is the most common mode of its inheritance [13]. The most notable genes related to bone remodeling are OTSC 1-8, CD46, tumor growth factor β1 (TGF-β1), collagen 1A1 (COL1A1), bone morphogenetic protein (BMP) 2, and BMP 4 [1, 17]. There is a proposed association of class I major histocompatibility complex with otosclerosis; some studies support this while others have failed to replicate their association [18]. Studies have shown the role of these genes in the development of otosclerosis, while other genes, although associated, their role in the development of this disease is still unspecified [9]. These genes and their products are associated with pathological manifestations. A study has shown a significant association between the disease and the COL1A1 gene, product being type I collagen protein [19]. Other genes and their product also have an autoimmune and inflammatory role.

Autoimmunity and inflammation

A study showed the presence of an autoantibody against type II collagen in the serum of otosclerotic patients [20]. TGF-β1 cytokine has been implicated and studied in the pathogenesis of otosclerosis [21]. Increased expression of tumor necrosis factor α (TNF-α) is seen, which leads to osteoclast activation and bone resorption [9]. There is an association of BMP isoforms with the remodeling in active otosclerosis [22]. Several studies have shown and reported the involvement of different autoantibodies, inflammatory cytokines, and growth factors in the pathogenesis of otosclerosis.

Role of hormones in otosclerosis

Otosclerosis occurs two to three times more frequently in females than in males, and symptoms manifestation occurs more frequently during or after pregnancy, which indicates endocrinal factors in its pathogenesis [23]. Angiotensin II of the renin-angiotensin-aldosterone system (RAAS) influences the secretion of TNF-α [1]. There is a well-known role of parathormone, estrogen, and progesterone in bone remodeling, which is present in otosclerotic bone samples [9, 24]. There is a naturally occurring balance between receptor activator of nuclear factor κ Β ligand (RANK-L) and osteoprotegerin (OPG) that plays an important role in balanced bone remodeling, which is under the influence of estrogen and prolactin [24]. Estrogen reduces the response of osteoclasts to RANK-L, and apoptosis of osteoclast increases; likewise, increased prolactin level decreases OPG and increases RANK-L production [24]. These studies report the role of the different hormones in the pathogenesis of bone remodeling. An imbalance can lead to abnormal bone remodeling, thus otosclerosis.

Viral infection: role of persistent measles virus infection

The measles virus is from the Paramyxoviridae family, which has a non-segmented, negative-sense RNA genome. Measles virus infection presents as fever and maculopapular rash. Other clinical manifestations include cough, coryza, and conjunctivitis. The widespread use of measles vaccines has decreased its incidence, morbidity, and mortality [7]. Complications of measles include neurological diseases such as acute disseminated encephalomyelitis, measles inclusion body encephalitis, and subacute sclerosing panencephalitis. Other complications are keratoconjunctivitis, stomatitis, laryngitis, diarrhea, pneumonia, and otitis media [7]. Measles can also complicate pregnancy leading to adverse pregnancy outcomes [7]. It can affect multiple organ systems where death can also occur [7]. In addition to these complications, persistent measles virus infection has been postulated in the etiopathogenesis of otosclerosis. Otosclerosis is an autoimmune inflammatory disorder related to abnormal bone remodeling of the human otic capsule with yet inexplicable etiopathogenesis. Many propositions are put forward to explain the etiology of otosclerosis. One of them is a persistent measles virus infection [11]. This review article was performed to study the association between persistent measles virus infection and otosclerosis. After a careful review of all the selected studies, we believe that there are a lot of etiological factors that can lead to the causation of otosclerosis. However, to firmly say if measles virus infection is one of many causes of otosclerosis is still undetermined.

We found out that the majority of observational studies detected measles virus RNA in otosclerotic stapes by different methods. Several observational studies used various methodologies like reverse transcription polymerase chain reaction (RTPCR), reverse transcription-quantitative polymerase chain reaction (RTQPCR), and glyceraldehyde 3-phosphate (GADP) for detecting messenger RNA (mRNA) of measles virus from otosclerotic stapes sample and control samples [2, 11, 12]. Measles virus antigen and antibodies against measles virus were also detected in other studies. There were more technical methodologies in various studies employed to detect measles virus-related genomes, antigen, antibodies, and tissue growth factors from samples.

In our study, we figured four major groups of researchers who contributed studies on measles virus and otosclerosis. Karosi T, along with his colleagues, performed 11 observational studies, one review article, and one systematic review. All of his 11 observational studies detected measles virus mRNA in most clinical and histological otosclerotic stapes footplate. His notable observational findings in which he detected measles virus mRNA were in 62 out of 102, 175 out of 261, 99 out of 154, 20 out of 34 otosclerotic stapes samples [25-28]. His other articles supported the same, and he proposed the possible role of persistent measles virus infection in the causation of otosclerosis [1, 29]. Niedermeyer HP et al. conducted six observational studies and three review articles. In all of his observational studies, he detected the presence of measles virus mRNA in most otosclerotic stapes. His notable observational findings in which he detected measles virus mRNA were in 32 out of 40, 79 out of 95, 13 out of 14 otosclerotic stapes samples, and in a sample size of 1352 with a p-value of 0.012 [30-33]. His review article also supported the same [14, 34, 35]. Arnold W et al. and McKenna MJ et al. conducted five and four observational studies, respectively. They also detected measles virus RNA or their antigen or antibodies against them in superior numbers of otosclerotic study samples. The findings in observational studies conducted by McKenna MJ et al. in which he detected measles virus mRNA were eight out of 11 and four out of four otosclerotic stapes samples [36, 37]. The findings in observational studies conducted by Arnold W et al. in which he detected measles virus mRNA were 15 out of 15, 19 out of 19 otosclerotic stapes samples [38, 39]. We also observed a decline in the incidence of otosclerosis and the shift of the age of incidence to the older population (54 years). This was largely due to widespread measles vaccination as depicted in few studies in Europe including a separate study in Germany, from separate observational studies by Niedermeyer HP et al. and Arnold W et al. [6, 31, 33, 40]. A recent review article by Liktor B et al. in 2018 cited the demonstration of measles virus and transforming growth factor-beta 1 (TGFβ1) gene that is a trigger factor in the etiopathogenesis of otosclerosis [41].

Table 3 summarizes the studies which showed an association between Otosclerosis and Persistent Measles virus infection from selected data for this review article.

**Table 3:** Summary of the studies that support the association between persistent measles virus infection and otosclerosis from selected data for this review article

Author Name/Year	Study Design	Study Results
Liktor B et al. [41], 2018	Review Article	This study concluded the measles virus as one of the possible etiologic factors.
Schrauwen I et al. [17], 2010	Review Article	This article concluded environmental factors play a role in the development of otosclerosis in addition to genetics, fluoride, and measles being the possible environmental factor.
Sziklai I et al. [42], 2009	Observational Study	This study detected the measles virus only in otosclerotic stapes footplate.
Karosi T et al. [1], 2009	Systematic Review	This study concluded various factors play a role in the etiopathogenesis of otosclerosis, persistent measles virus infection being one of them.
Karosi T et al. [43], 2008	Observational Study	This study detected measles virus RNA only in otosclerotic stapes.
Niedermeyer HP et al. [14], 2008	Review Article	This study concludes a strong association between measles virus and otosclerosis and proposes the measles virus as one of the possible triggers for otosclerosis.
Babcock, TA et al. [44], 2018	Review Article	This study supports the proposed autoimmune role of chronic measles virus infection in the initiation of otosclerosis.
McKenna MJ et al. [45], 2007	Review Article	This study concluded the evidence of the role of the measles virus in otosclerotic cases.

Despite all this evidence of the association, few observational studies have failed to obtain the association between persistent measles virus infection and otosclerosis. A recent observational study by Crompton M et al. in 2019 did not show any relationship. Another observational study by Singh MP et al. (2005) could only detect immunoglobulin M (IgM) antibodies against measles in 18.1% of his sample study, IgM antibodies against varicella zoster virus (VZV) could be detected in 4.5% of the same sample [46]. With these findings, he concluded that otosclerosis is not associated with a systemic viral infection. Flores-García ML et al. (2018) conducted an observational study that detected measles virus mRNA in only 3.3% (three of 93) of his study sample [47]. Separate observational studies by Komune N et al. (2012) and Grayeli AB et al. (2000) could not detect the presence of measles virus in the majority of their study sample. These observations could not assure the hypothesis of persistent measles virus infection in the causation of otosclerosis [11, 12].

Table 4 summarizes the studies which did not show an association between otosclerosis and persistent measles virus infection from selected data for this review article.

**Table 4:** Summary of the studies that do not support an association between persistent measles virus infection and otosclerosis from selected data for this review article

Author Name/Year	Study Design	Study Results
Komune N et al. [11], 2012	Observational Study	This study concluded no evidence of persistent measles virus infection in otosclerotic footplates in Japanese patients.
Grayeli AB et al. [12], 2000	Observational Study	This study could not detect the measles virus in a large number of otosclerotic samples. Based on this finding, this study could not confirm the theory of persistent measles virus infection in the etiopathogenesis of otosclerosis
Crompton M et al. [48], 2019	Observational Study	This study could not detect any association between otosclerosis and measles virus infection.
Flores-García ML et al. [47], 2018	Observational Study	Only three out of 93 patient samples tested positive for measles virus gene components. None of the samples contained measles virus RNA. This study did not support the hypothesis of the measles virus as a possible trigger of otosclerosis.

One large observational study by Niedermeyer HP et al. with a sample size of 1351 showed strong support for the involvement of the measles virus in the causation of otosclerosis while another sizeable observational study by Crompton M et al. in 2019 with a sample size of 657 did not show any relationship [33, 48].

Persistent infection by the measles virus has been postulated in many chronic diseases with an inflammatory component, such as otosclerosis, multiple sclerosis, Paget’s disease, and Crohn’s disease [12]. In this particular case, many studies have detected the presence of measles virus RNA in otosclerotic stapes in large samples, which are statistically significant. However, there is inconsistent and nonreproducible detection of measles virus in other studies even with highly sensitive tests and large samples [12].

Several research articles describe chronic measles virus infection as one of the main critical factors in the development of otosclerosis in an individual. A total of 33 observational studies from 1986 to 2015, 13 review articles, and a systematic review found the same. On the contrary, a total of five observational studies from 2000 to 2019 reports no evidence of the association.

We analyzed that even with highly sensitive methods, the measles virus is detected in a large number of otosclerotic samples. However, few other studies have failed to show their presence. This observation questions about the hypothesis however these conflicting results make the role of measles virus in otosclerosis a big mystery. Since so many studies favour the association, it can be hypothesized that persistent measles virus infection has to be among one trigger for otosclerosis.

Treatment discussion

Surgery, either stapedotomy or stapedectomy, is the chief curative approach in the management of otosclerosis to cure hearing loss. Sodium fluoride has also been tried to reduce the severity of the disease. However, its use in the clinical scenario is still lacking. Few studies show that it can reduce the degree of hearing loss while other studies do not support this association [17]. Due to its complicated etiopathogenesis, which includes bone remodeling abnormality, autoimmunity and inflammation, anti-inflammatory and anti-osteoporotic drugs are considered in treating this condition. Drugs like non-steroidal anti-inflammatory drugs (NSAIDs) (e.g., indomethacin), TNF biologics (e.g., infliximab), bisphosphonates, calcitonin, supplemental vitamin D and short term recombinant OPG may all have a beneficial effect on the disease process [1]. Surgical option in the treatment of otosclerosis is well studied and is the primary method of treatment of otosclerosis, but the role of pharmacotherapy in the degree of improvement in the disease severity is still lacking, and their use is less in clinical practice. Many studies cited the association of otosclerosis and measles virus, but there have been no therapeutic consequences yet that can be derived out of this association [41]. The widespread use of the vaccine against measles has decreased the incidence of otosclerosis and increased the mean age of onset for otosclerosis [6].

## Conclusions

The objective of our review article was to study the etiopathogenesis of otosclerosis and its association with persistent measles virus infection. The goal was to study the impact of persistent measles virus infection on triggering the disease process and therapeutic consequences if any. Otosclerosis is an autoimmune inflammatory disorder related to abnormal bone remodeling of the human otic capsule, which has complex etiopathogenesis attributed to genetics, autoimmunity, viral infection, inflammation, hormonal factor, environmental factor, and disturbed bone metabolism. The majority of the literature review supported the evidence of the presence of the measles virus component in the otosclerotic stapes samples even after the elimination of measles virus infection. Measles virus infection may have the amplitude to initiate a pathological process, which in the presence of other factors like autoimmunity and genes implicated to play a significant role, can lead to the onset of otosclerosis. Due to this association, the widespread use of a vaccine to prevent measles has decreased the incidence of otosclerosis fortuitously. This relationship also opens up a scope for the research in related clinical pharmacotherapy for useful treatment advances. Though it is hard to say that the mere existence of the measles virus part in otosclerotic samples can lead to otosclerosis as other studies contradict this theory. Nevertheless, more studies are needed to probe the role of persistent measles virus infection in the etiopathogenesis of otosclerosis.
